# Protein Kinase A Dependent Phosphorylation of Apical Membrane Antigen 1 Plays an Important Role in Erythrocyte Invasion by the Malaria Parasite

**DOI:** 10.1371/journal.ppat.1000941

**Published:** 2010-06-03

**Authors:** Kerstin Leykauf, Moritz Treeck, Paul R. Gilson, Thomas Nebl, Thomas Braulke, Alan F. Cowman, Tim W. Gilberger, Brendan S. Crabb

**Affiliations:** 1 Macfarlane Burnet Institute for Medical Research & Public Health, Melbourne, Victoria, Australia; 2 Bernhard Nocht Institute for Tropical Medicine, Department of Molecular Parasitology, Hamburg, Germany; 3 Walter & Eliza Hall Institute of Medical Research, Melbourne, Victoria, Australia; 4 Department of Biochemistry, Children's Hospital, University Medical Center Hamburg-Eppendorf, Hamburg, Germany; 5 M.G. DeGroote Institute for Infectious Disease Research, McMaster University, Hamilton, Ontario, Canada; 6 The University of Melbourne, Victoria, Australia; 7 Monash University, Victoria, Australia; Albert Einstein College of Medicine, United States of America

## Abstract

Apicomplexan parasites are obligate intracellular parasites that infect a variety of hosts, causing significant diseases in livestock and humans. The invasive forms of the parasites invade their host cells by gliding motility, an active process driven by parasite adhesion proteins and molecular motors. A crucial point during host cell invasion is the formation of a ring-shaped area of intimate contact between the parasite and the host known as a tight junction. As the invasive zoite propels itself into the host-cell, the junction moves down the length of the parasite. This process must be tightly regulated and signalling is likely to play a role in this event. One crucial protein for tight-junction formation is the apical membrane antigen 1 (AMA1). Here we have investigated the phosphorylation status of this key player in the invasion process in the human malaria parasite *Plasmodium falciparum*. We show that the cytoplasmic tail of *P. falciparum* AMA1 is phosphorylated at serine 610. We provide evidence that the enzyme responsible for serine 610 phosphorylation is the cAMP regulated protein kinase A (*Pf*PKA). Importantly, mutation of AMA1 serine 610 to alanine abrogates phosphorylation of AMA1 *in vivo* and dramatically impedes invasion. In addition to shedding unexpected new light on AMA1 function, this work represents the first time PKA has been implicated in merozoite invasion.

## Author Summary

The invasion of host cells by zoites of the phylum apicomplexa is an active event that is powered by the parasite invasion machinery. It can be divided in several distinct steps that involve binding to the host cell, reorientation and tight junction formation that are accompanied by sequential secretion of specialised organelles that store proteins involved in these events. A great number of proteins are now known to be involved in invasion but how the invasion process is regulated remains obscure. Recently, phosphorylation of some proteins with a defined function in invasion like GAP45, MTIP and AMA1 were reported and provided the first insight into putative regulation mechanism of invasion. Using mutational analysis we now demonstrate that AMA1 is phosphorylated in the cytoplasmic domain at serine 610 in a cAMP dependent manner and that mutation of S610 dramatically reduces the efficiency of invasion into erythrocytes. We identified protein kinase A (*Pf*PKA) as a late transcribed kinase that is responsible for phosphorylating AMA1 at this specific residue. This work describes for the first time PKA signalling being implicated in merozoite invasion, providing a new avenue for understanding the initiation and regulation of invasion. Significantly also, the PKA-AMA1 pathway defines a promising new and validated drug target for therapeutic intervention.

## Introduction

Malaria is one of the most devastating infectious diseases of mankind and is a leading cause of morbidity and mortality in tropical and sub-tropical regions where 40% of the world's population live. The most pathogenic species that infects humans is *Plasmodium falciparum* and in 2002 it was estimated that out a total of 515 million clinical cases, 2–3 million were fatal [1]. Central to malarial pathogenesis is the large-scale invasion of red blood cells (RBCs) by *Plasmodium* parasites. The invasive merozoite forms of the parasite infect RBCs via a complex multi-step process involving sequential receptor-ligand interactions and signal transduction events (reviewed in [2]). Merozoite invasion is an intense area of investigation by many groups as it is a point in the parasite lifecycle that is particularly vulnerable to immune and drug intervention. While signalling within the parasite, particularly that triggered by calcium, is known to be involved in RBC invasion, the specific nature of this process including the identity of the key molecular players remains largely a mystery. To address this we have been studying an essential transmembrane protein present on the invasive merozoite surface, apical membrane antigen 1 (AMA1). AMA1 is one of the most promising blood-stage malaria vaccine candidates and is amongst the best studied of the ∼5000 *Plasmodium* proteins.

In *P. falciparum*, AMA1 is synthesised during merozoite development towards the end of the blood stage cell cycle and is stored in apical secretory organelles called micronemes [3], [4]. It is a type I integral membrane protein of 83 kDa with a large N-terminal ectodomain, a single transmembrane domain near the C-terminus and a small cytoplasmic tail of 56 amino acids (*Pf*AMA1_83_) [5], [6]. Before schizont rupture the N-terminal prosequence is cleaved resulting in a 66-kDa form (*Pf*AMA1_66_) that translocates from the micronemes to the merozoites surface [7]. In a second proteolytic processing step during invasion the bulk of the ectodomain is shed quantitatively as 44-kDa and 48-kDa fragments by the membrane bound subtilisin-like protease *Pf*SUB2 leaving a 22-kDa transmembrane fragment that is taken into the newly invaded RBC [8], [9]. AMA1-specific antibodies and peptides block invasion at a step after the long-distant primary contacts between parasite and host cell have occurred but prior to the close interactions seen during tight junction formation [10]–[12]. Some functional insights of antibody-based inhibition were gained from using the monoclonal antibody 4G2 that targets an epitope adjacent to the conserved hydrophobic trough in the ectodomain [13]–[15]. This antibody binding led to the abrogation of AMA1 interaction with rhoptry neck proteins (RONs, [14]) that was previously established as important part of the tight junction [16], [17]. Apart from the critical interactions of the ectodomain the cytoplasmic tail of AMA1 has also been shown to be essential for invasion [18]. Mutational analysis hinted an important role for tail phosphorylation in the invasion process.

In this paper we show that the *Pf*AMA1 tail is phosphorylated at a specific serine residue (S610) and that the enzyme responsible for this event is the parasite-encoded protein kinase A (*Pf*PKA). Moreover, we show that S610 plays a crucial role for AMA1 function and parasite invasion. This phosphorylation event has implications for understanding the regulation of invasion, for the function of AMA1 and for the development of new therapeutic approaches.

## Results

### The cytoplasmic domain of AMA1 is phosphorylated by a cAMP dependent kinase present in schizont and merozoite lysates

**Figure 1:**
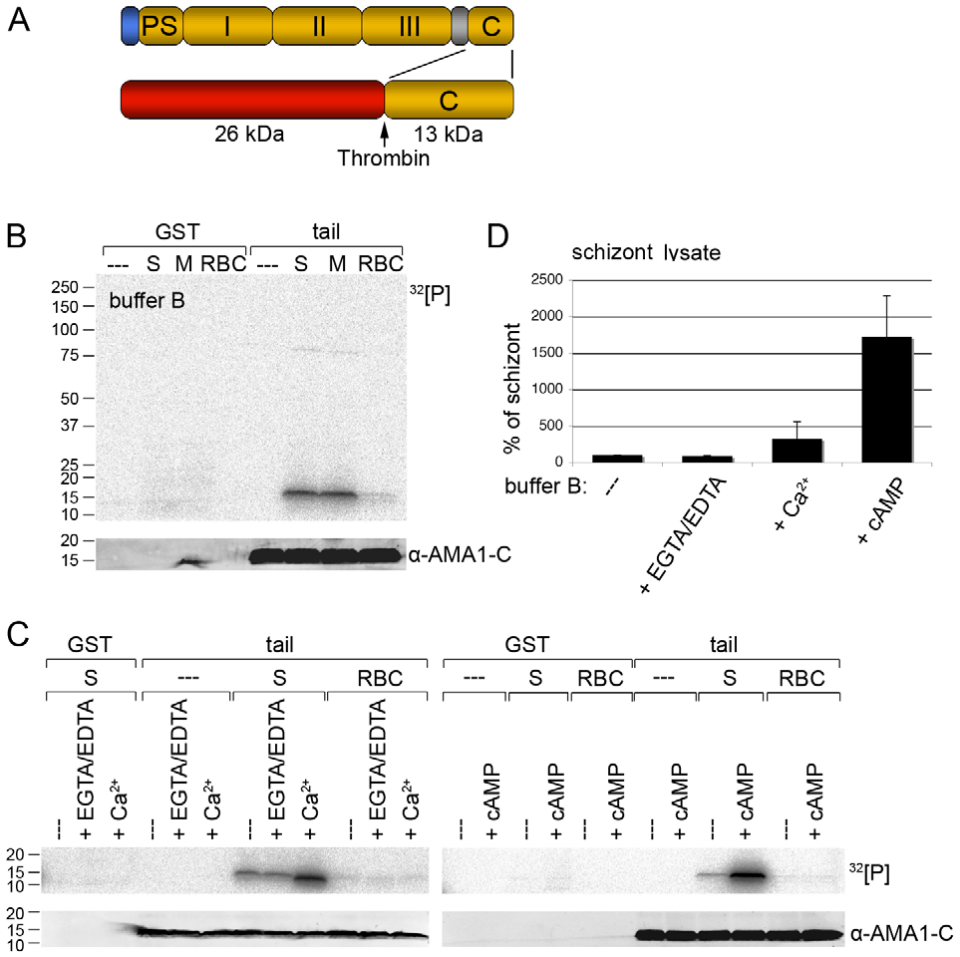
Recombinant AMA1 C-terminal tail is phosphorylated *in vitro* by *P. falciparum* (3D7 line) parasite lysates in a calcium and cAMP dependent manner. (**A**) Schematic representation of AMA1 and the GST-fusion protein used. Signal peptide (blue), prosequence (PS), ectodomains I, II & III, transmembrane domain (grey), cytoplasmic tail (C) and thrombin cleavage site are indicated. (**B, C**) Auto-radiographs showing phosphorylation of recombinant AMA1 tail by parasite lysates in the presence of 1.5 mM EGTA/1 mM EDTA or 2 mM CaCl_2_ or 1 µM cAMP. The AMA1 tail was incubated with schizont (S), merozoite (M) or red blood cell (RBC) lysates and ^32^[P]-γ -ATP. After washing the GST part was cleaved off with thrombin. As a loading control the membrane was probed with an anti-AMA1 antibody detecting the AMA1 tail. Molecular sizes are indicated on the left. (**D**) Quantitation of signal intensities in panel C with Image Gauge software. In the absence of additional EGTA/EDTA or cAMP the strength of the phosphorylation signal in untreated schizont lysate was set to 100% and all other signals are relative to that. The numbers of experimental replicates in *in vitro* phosphorylation assays are found in the Supplementary data (Text S1). Error bars correspond to standard deviation.

To investigate if the cytoplasmic domain of AMA1 is phosphorylated by parasite kinases we generated a glutathione S-transferase (GST) fusion protein of the AMA1 cytoplasmic tail (Figure 1A) and performed *in vitro* phosphorylation assays with *P. falciparum* 3D7 lysates. Autoradiography of the AMA1 tail resolved by SDS-PAGE indicated it was specifically phosphorylated to comparable amounts by schizont and merozoite lysates (Figure 1B). Control reactions with non-infected RBC lysate gave only a background signal, indicating that the AMA1 tail was phosphorylated by parasite kinases rather than RBC kinases (Figure 1B). As loading controls the membrane was probed with an anti-AMA1 antibody that specifically recognised the AMA1 tail. No signal can be detected for the GST proteins because the antibody used was specific for the AMA1 tail only (Figure 1).

Invasion of RBCs by *Plasmodium* is known to involve calcium ion (Ca^2+^) fluxes [19] which might trigger AMA1 phosphorylation. To address this, calcium ions in parasite lysates were chelated by EGTA/EDTA before incubation with the AMA1 tail. Conversely, to increase the calcium concentration in the assay 2mM CaCl_2_ was added to the buffer. Whereas EGTA/EDTA had little effect on the intensity of the phosphorylation signal compared to the untreated sample (Figure 1C, left panel and Figure 1D), the addition of Ca^2+^ to the buffer resulted in a 3 fold increase of the phosphorylation signal (Figure 1C, left panel and Figure 1D).

In addition to Ca^2+^, cAMP is also a common second messenger and has been shown to be involved in parasite development during the asexual blood stages [20], [21]. Thus, we tested if cAMP had an effect on AMA1 tail phosphorylation. As shown in Figure 1C (right panel) the addition of cAMP to the *in vitro* phosphorylation assay led to a dramatic 17-fold increase of AMA1 tail phosphorylation (Figure 1D), which indicated an involvement of the protein kinase A (PKA) in the phosphorylation event.

### The recombinant AMA1 tail is phosphorylated on residue S610

**Figure 2:**
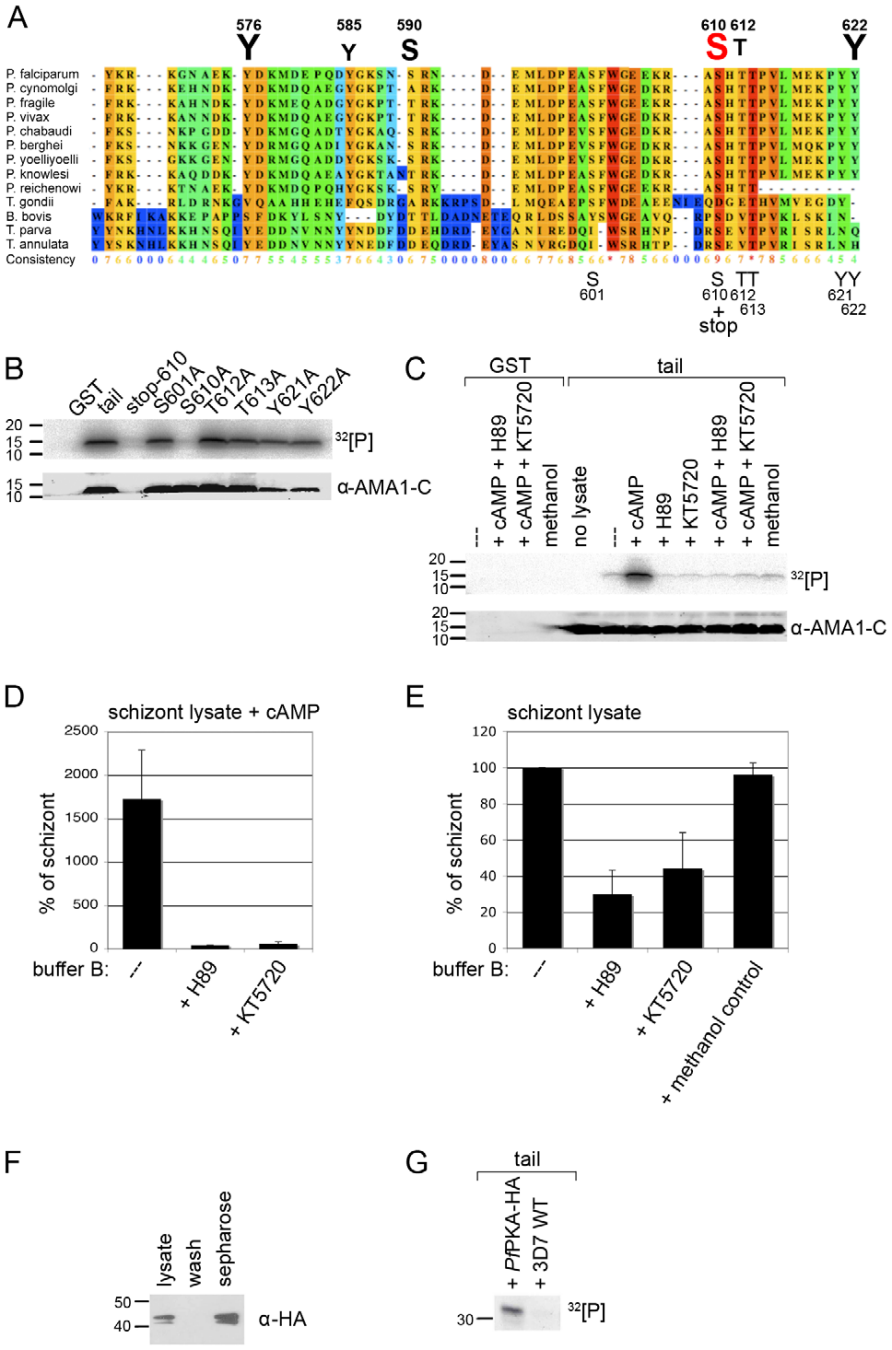
*Pf*PKA phosphorylates the AMA1 tail exclusively on residue S610. (**A**) Multiple alignment of AMA1 C-terminal cytoplasmic domains including protein sequences of nine different *Plasmodium* species, *T. gondii*, *B. bovis* and two *Theileria* species. The conservation is scored and colour coded by PRALINE (www.ibi.vu.nl). Amino acids predicted to be phosphorylated in *P. falciparum* AMA1 by NetPhos (www.cbs.dtu.dk/services/NetPhos) are scaled up in font size according to their relative predicted probabilities above the alignment and numbered regarding the protein sequence. As shown below the alignment the GST-fusion protein of the AMA1 tail was mutated at residues S601, S610, T612, T613, Y621 and Y622, respectively. (**B, C**) SDS-PAGE and radiograph of phosphorylation samples. GST-fusion proteins of the AMA1 tail and mutants were incubated with schizont lysate and ^32^[P]- γ -ATP. As a loading control the membrane was probed with an anti-AMA1-C antibody. (**C**) H89 (50 µM) and KT5720 (10 µM), two inhibitors to PKA reverse the effect of cAMP (1 µM) on AMA1 tail phosphorylation. (**D, E**) Quantitation of signal intensities in (**C**) with Image Gauge software. The phosphorylation signal strength in untreated schizont lysate was set to 100% and all other signals were relative to that. The numbers of experimental replicates in *in vitro* phosphorylation assays are found in the Supplementary data (Text S1). Error bars correspond to standard deviation. (**F**) *Pf*PKA-HA was immuno-precipitated from lysate of 3D7*_Pf_*_PKA-HA_ transgenic parasites using protein-G-sepharose. *Pf*PKA-HA was detected by Western Blot analysis using anti-HA antibodies and parasite lysate, washed fraction or sepharose beads. (**G**) SDS-PAGE and radiograph of *in vitro* phosphorylation of AMA1-GST using either protein-G-sepharose incubated with 3D7*_Pf_*_PKA-HA_ or with wild type (WT) parasites in the presence of ^32^[P]- γ -ATP.

A multiple alignment of 13 AMA1 protein sequences using the software PRALINE (www.ibi.vu.nl) showed that the C-terminal cytoplasmic domain of apicomplexan AMA1 is highly conserved among different *Plasmodium* species as well as other apicomplexans (*Toxoplasma gondii*, *Babesia bovis*, *Theileria parva*, *Theileria annulata*) suggesting a common function during host cell invasion. Additionally, the AMA1 tails contain several potential phosphorylation sites with six amino acids being predicted as phosphorylation sites in *Pf*AMA1 (www.cbs.dtu.dk/services/NetPhos) (Y576, Y585, S590, S610, T612, Y622, Figure 2A).

The dependence on cAMP suggested that parasite-encoded PKA (PFI1685w), the only apparently recognisable cAMP dependent kinase expressed at this stage of the life cycle [22], was responsible for the observed phosphorylation. Consistent with that, the NetPhosK program (www.cbs.dtu.dk/services/NetPhosK) predicted that residue S610 is phosphorylated by PKA, showing the highest score when compared to all the other serines, threonines and tyrosines in the AMA1 tail. To establish if the prediction of S610 phosphorylation by *Pf*PKA was correct, site-directed mutagenesis was performed. Firstly, a GST-fusion protein mutant containing a stop codon at position S610 was generated (Figure 2A) that lacks the highly conserved C-terminus with its putative phosphorylation sites including the S610. *In vitro* phosphorylation assays with 3D7 schizont lysates indicated this mutant was not phosphorylated in the presence of cAMP (Figure 2B), suggesting that none of the less conserved proximal predicted phosphorylation sites Y576, Y585, S590 were involved in this phosphorylation event or that if these sites are phosphorylated their phosphorylation is dependent on the presence of serine 610 or residues downstream of S610. Although tyrosine kinases are apparently absent in the parasite's genome [23] two predicted tyrosines were included in the mutagenic analyses as controls. The band for the stop mutant is missing in the loading control since the anti-AMA1 antibody detects a peptide C-terminally of S610stop (Figure 2B). Secondly, to verify that S610A is responsible for protein phosphorylation, we exchanged the remaining phosphorylation sites (including S601) and subjected these mutant AMA1s to *in vitro* phosphorylation. Only the stop mutant and S610 lacked a phosphorylation signal suggesting that S610 is either the only residue being phosphorylated under the given conditions or S610 phosphorylation enables the phosphorylation of other sites in the AMA1 tail (Figure 2B).

### *Pf*PKA phosphorylates AMA1 S610

To confirm the involvement of *Pf*PKA as the phosphorylating kinase, two PKA inhibitors, H89 and KT 5720, were tested for their ability to block phosphorylation of the AMA1 cytoplasmic domain. These compounds are competitive antagonists of ATP's access to a binding pocket on the catalytic subunit of PKA and H89 has been used in *Plasmodium* parasites previously where it appears to affect the cell cycle and arrest proliferation during schizogany [20], [21]. To determine biologically relevant concentrations of PKA inhibitors to use in the *in vitro* phosphorylation assays, the growth inhibitory effects of the drugs were measured in live cultures. These indicated that the IC90 for H89 and KT 5720 was about 50 µM and 10 µM, respectively (data not shown). In the *in vitro* phosphorylation assays, both compounds completely blocked the stimulatory effect of cAMP upon the phosphorylation of the recombinant AMA1 tail indicating potent inhibition of PKA stimulation (Figure 2C and 2D). In the absence of additional cAMP, H89 and KT 5720 decreased AMA1 tail phosphorylation by 70% and 55% respectively in *in vitro* phosphorylation assays using 3D7 schizont lysates (Figure 2C and 2E). Although we can't completely rule out that the inhibition of cAMP stimulated kinase activity is due to methanol, the solvent of KT5720, rather than KT5720 itself, it is very unlikely for two reasons: firstly, both inhibitors show similar inhibitions, even if only KT5720 is dissolved in methanol, whereas H89 is dissolved in H_2_O. Secondly, methanol per se had no effect on the outcome of the *in vitro* phosphorylation assay in the absence of additional cAMP as shown in Figure 2C and 2E. Although H89 and KT5720 have been used extensively in animal cell PKA studies for many years some doubts about the specificity of these compounds for PKA, especially at the used concentrations, remain and they may have effects on other kinases such as PKB [24]. We can therefore not be entirely sure that the background (unstimulated) level of AMA1 tail phosphorylation is completely due to PKA since other kinases may also be inhibited.

To further validate *Pf*PKA as an AMA1 phosphorylating kinase, we over-expressed HA-tagged *Pf*PKA catalytic subunit in late blood stages. Transgenic expression was verified by either Western Blotting or IFA using anti-HA antibodies (Figure S1). The antibodies detected a double band at around 43 kDa corresponding well with the theoretical molecular weight of the fusion-protein of 42.5 kDa. The doublet might represent a phosphorylated form of *Pf*PKA as has been described for other PKA proteins [25]. *In vivo* activation of PKA in *Schizosaccharomyces pombe* requires threonine phosphorylation at its activation loop and is dependent on PDK1 [26]. Subsequent immuno-precipitation of the HA-tagged *Pf*PKA from 3D7*_Pf_*_PKA-HA_ parasites allowed the purification of the *Pf*PKA-HA (Figure 2F). Late stage specific over-expression of the catalytic subunit of *Pf*PKA had no effect on parasite growth rate (data not shown). This purified *Pf*PKA readily phosphorylated the recombinant AMA1 tail (Figure 2G). Taken together, the *in vitro* data suggest that cAMP triggers AMA1 tail phosphorylation on residue S610 by *Pf*PKA.

### Phosphorylation of native AMA1 and the importance of S610 for RBC invasion

**Figure 3:**
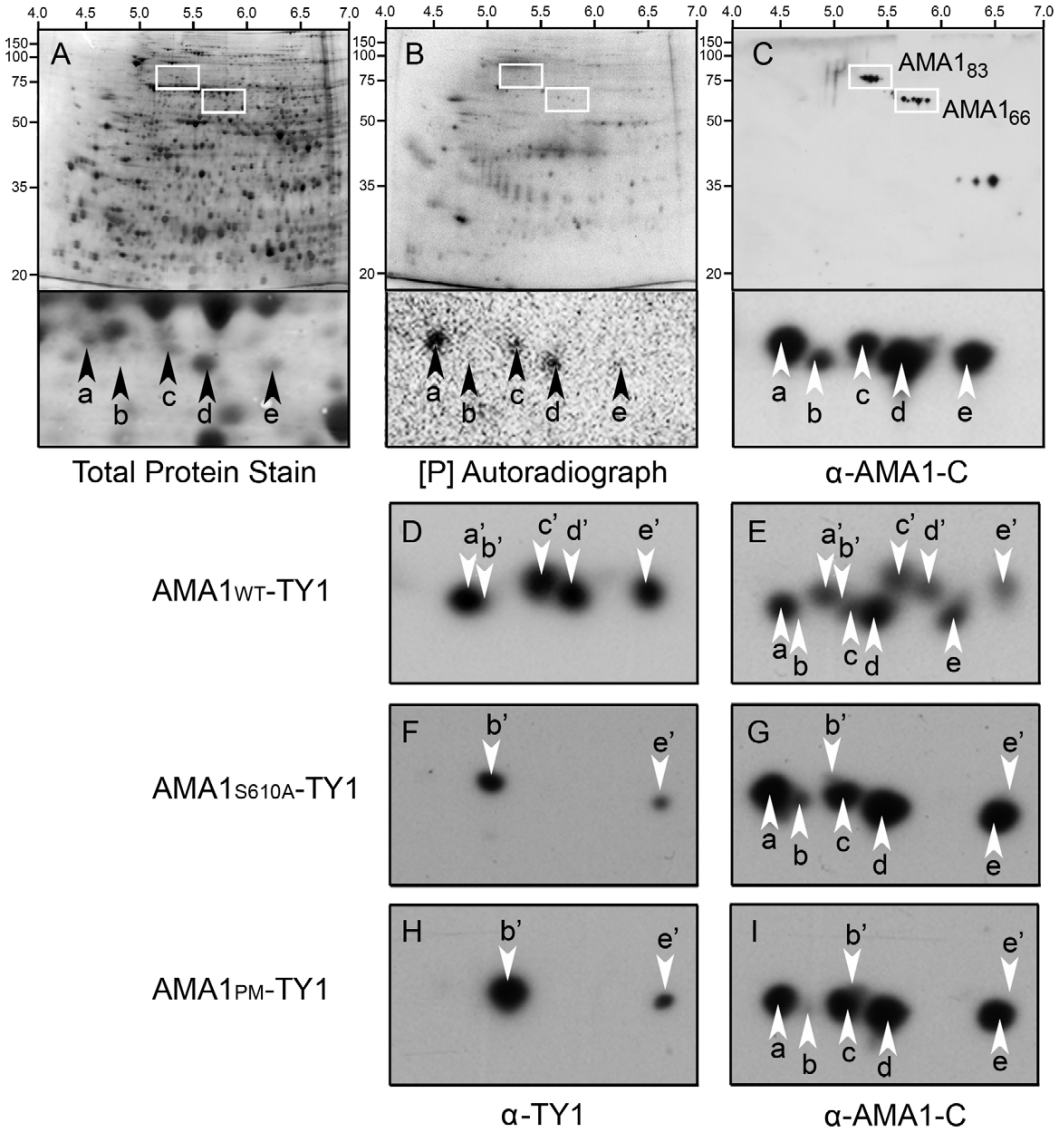
Two dimensional gel analyses confirm that *P. falciparum* AMA1 is phosphorylated at S610 *in vivo*. *P. falciparum* proteins were metabolically labelled with ^32^[P], detergent extracted and resulting 2DE blots developed using Deep Purple stain to visualize total protein (**A**). Autoradiography detected *in vivo* phosphorylated proteins (**B**), and Western Blotting with an anti-AMA1 antibody detected the AMA1 tail (**C**). Isoelectric point and molecular sizes are indicated on the top and left. 2D spots corresponding to the 83 kDa precursor (AMA1_83_) or post-translationally processed ∼66 kDa AMA1 fragment (AMA_66_) are highlighted by bounding boxes, respectively. Magnification of the AMA1_66_ region reveals a series of five discrete ^32^[P]-labelled (a, c, d) or -unlabelled (b, e) protein spots recognized by anti-AMA1 antibody (arrows). (**D-I**) all show ∼66 kDa species of AMA1 separated by 2DE and analysed by Western Blot. 3D7 parasites either expressed a transgenic TY1-tagged wild type W2mef AMA1 (AMA1-WT-TY1; **D, E**), or mutant forms where the W2mef transgene carried the AMA1-S610A mutation (AMA1-S610A-TY1; **F, G**) or mutations at all putative phosphorylation sites in the cytoplasmic tail AMA1-PM-TY1; **H, I**). Blots were probed with a mouse monoclonal antibody against TY1 to detect transgenic epitope-tagged AMA1 followed and by an anti-AMA1 antibody against the AMA1 tail to detect both endogenously expressed 3D7 AMA1 as well as the W2mef transgenic species.

To investigate the phosphorylation status of native AMA1 we analysed mature schizont stage parasite extracts from 3D7 parental parasites by 2DE (Figure 3A-C). When analysed by Western Blotting using antibodies that recognise the C-terminus of AMA1, the 66 kDa AMA1 species was shown to be comprised of 5 distinct spots (termed “a-e” respectively with spot “a” being the most negatively charged and “e” the least) that separated on a basis of their isoelectric point. Three of these spots (a, c and d) incorporated radiolabelled phosphate indicating that these spots represent phosphorylated forms of AMA1 (Figure 3B).

To determine if S610 was phosphorylated *in vivo* the phosphorylation patterns of wild type AMA1 and the non-phosphorylatable S610A and PM (all potential sites mutated) mutants were compared by 2DE. Because previous studies had indicated these phosphorylation sites were essential for AMA1 function we used a complementation approach [18]. We generated 3D7 parasites episomally expressing the W2mef allelic form of AMA1 tagged at the C-terminus with the TY1 epitope to distinguish it from the endogenous 3D7 AMA1 protein. Three lines were created expressing either the W2mef wild type form (AMA1-WT-TY1), a form with the S610A mutation (AMA1-S610A-TY1) or another form with each potential phosphorylation site mutated (AMA1-PM-TY1) (Figure 3D-I). Samples were analysed by 2DE and Western Blot which were probed with either TY1 antibodies or polyclonal AMA1-C-terminal antibodies where indicated. It was apparent that the 66 kDa species in the AMA1-WT-TY1 line separates into 8 visible spots (Figure 3E) consistent with the expression of two different forms of AMA1 in this parasite line. The W2mef TY1 tagged spots, termed a'-e', were generally of slightly higher molecular weight than the untagged 3D7 species and their PIs were a little greater (Figure 3D,E). We assume 10 spots are present in this line but that 2 spots (b and b') are masked by other spots or are below detectable levels. Consistent with the masking of b', anti-TY1 antibodies detect spots a', c', d' and e' in AMA1-WT-TY1 parasites (Figure 3D). Strikingly, the banding pattern in AMA1-S610A-TY1 is much simpler with only 2 TY1-tagged spots observed, b' and e' (Figure 3F and G). Mutation of all potential phosphorylation sites in the AMA1 tail to alanine (including S610) gave an identical pattern to the S610A-only mutant (Figure 3H and I). The mutation of S610 to an alanine residue appears to have eliminated the presence of all phosphorylated species of AMA1. One possible scenario might be that spots a, c & d represent 3 separate phosphorylation sites on the protein with the modification of S610 as prerequisite for the phosphorylation of the other sites. Alternatively, all three spots could contain phosphorylated S610, but two of the three contain additional charge-modifying posttranslational modifications depending on S610 phosphorylation. Indeed, our data suggest that there are at least two forms that differ in charge for reasons other than phosphorylation (b & e). This data confirms a crucial role for S610 in the phosphorylation of AMA1.

**Figure 4:**
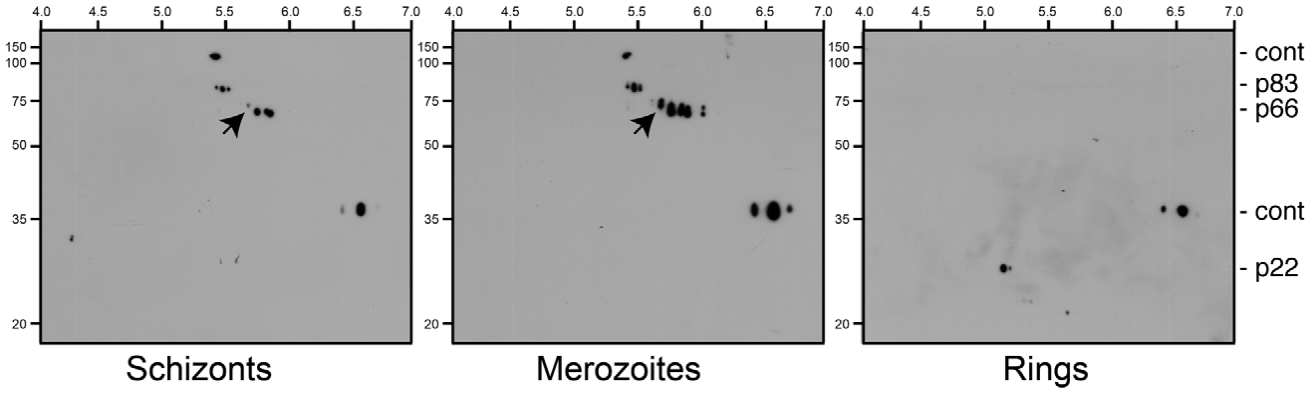
Maturation of phosphorylated AMA1 species. Schizont, merozoite and ring-stage parental 3D7 parasites were analysed from a sequential time-course experiment by two dimensional gel electrophoresis and Western Blotting. Blots were probed with C-terminal AMA1 tail antibodies. Bands representing 83 kDa, 66 kDa and 22 kDa AMA1 species as well as presumed cross-reacting contaminating species (cont) are indicated on the right. Isoelectric points and molecular weight markers are indicated on the top and left of the autoradiographs, respectively.

Due to the inability to obtain perfectly synchronous parasites it is difficult to precisely determine when S610 phosphorylation is occurring. However, to address this issue to some extent we prepared a time course experiment examining AMA1 species present in schizonts, merozoites and ring-stages (Figure 4). By Western Blotting 2DE gels with an antibody that recognises the C-terminal tail of AMA1 we show that the 66 kDa AMA1 species (a form that appears late in schizogony) has a more complex banding pattern in merozoites than it does in schizonts, most notably the appearance of a more negatively charged, apparently phosphorylated, species (arrowhead in Figure 4). This is in contrast to the 83 kDa precursor form of AMA1 that appears to change little in pattern between schizonts and merozoites. While, all phosphorylated species of the 66 kDa fragment can be detected in some schizont preparations (see Figure 3A-C for example), the time-course data shown in Figure 4 suggests that the bulk of secondary modifications of the AMA1 tail, including phosphorylation, occurs very late in blood stage development, perhaps even in the short-lived merozoites themselves. Interestingly, the cleaved C-terminal form of AMA1 in newly invaded ring-stage parasites reveals a much simpler pattern of secondary modifications, which could point towards - among other possibilities - a de-phosphorylation event of S610 during this stage (Figure 4). We caution that precise knowledge about the timing of AMA1 phosphorylation and its potential de-phosphorylation will require the generation of new reagents, most probably a functional S610 phospho-specific antibody.

**Figure 5:**
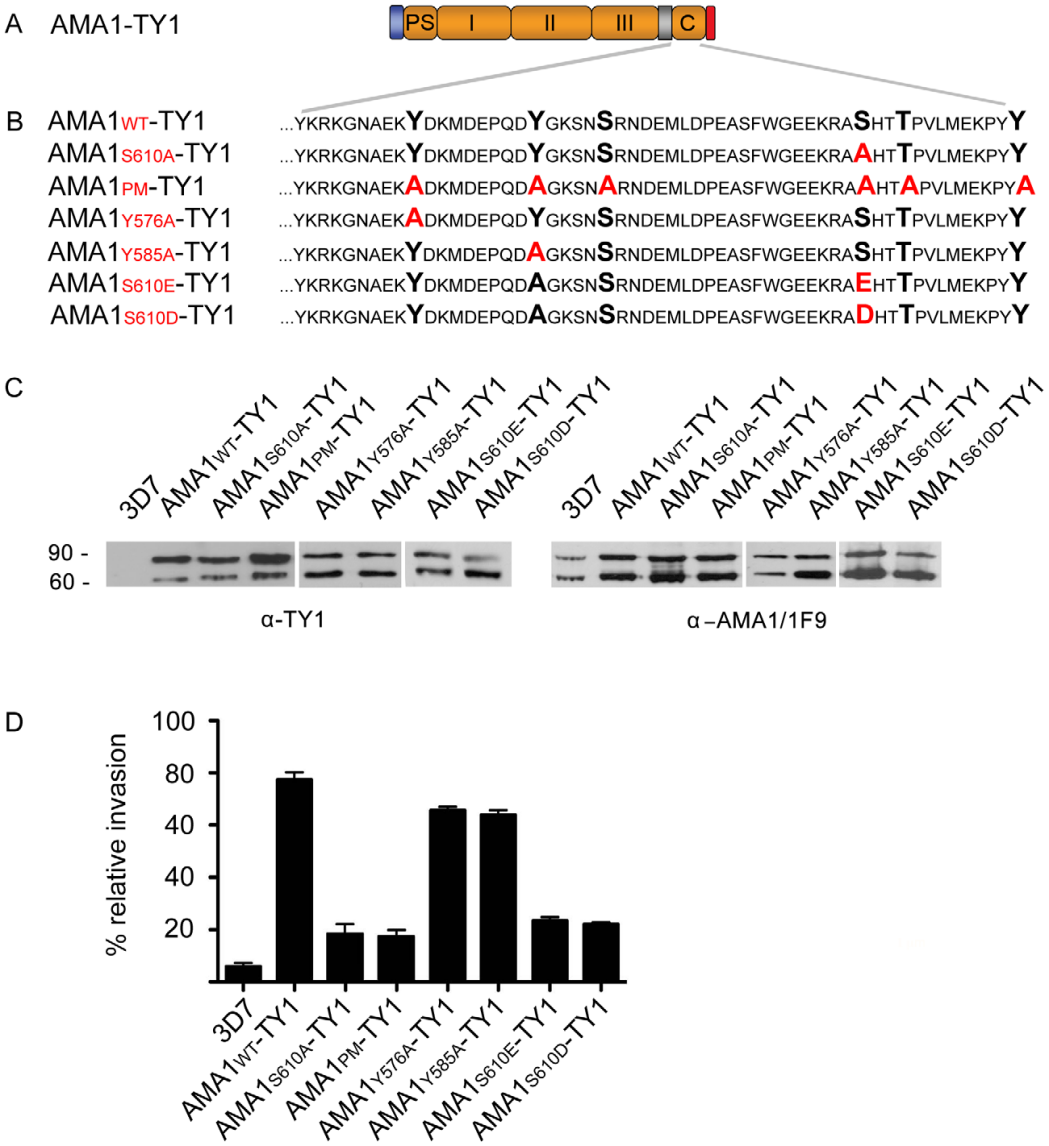
The *Pf*PKA phosphorylated residue S610 is required for efficient merozoite invasion. (**A**) Schematic representation of the ectopically expressed TY1-tagged AMA1. Signal peptide (blue), prosequence (PS), ectodomains I, II & III, transmembrane domain (grey), cytoplasmic tail (C) and TY1-tag (red) are indicated. (**B**) Mutations introduced into the cytoplasmic tail of W2mef-derived AMA1 are shown in red colour. (**C**) Expression of W2mef-derived AMA1-TY1 and native 3D7 AMA1 detected by Western Blot analysis using an anti-TY1 and anti-AMA1 (1F9) antibody, respectively (**C**, left panel). Whereas no AMA1-TY1 protein can be detected in 3D7 wild type parasites, a double band corresponding to AMA1_83_-TY1 and processed AMA1_66_-TY1 can be detected in AMA1-TY1-expressing parasites. (**C**, right panel) The endogenous 3D7 AMA1 is recognised by the anti-AMA1-1F9 antibody and shows identical proteolytic forms of AMA1 (AMA1_83_ and AMA1_66_). (**D**) Invasion inhibition assays using AMA1-TY1 expressing parasite strains. Assays were performed in the presence of 100 µg/mL R1 peptide and were performed in triplicate in three independent experiments. Error bars correspond to standard deviation. 3D7 and AMA1-WT-TY1 served as control.

We next tested the functional consequences of S610A mutation *in vivo*. To do this we used a similar complementation approach as previously described [18], [27]. Briefly, full length AMA1 bearing a C-terminal TY1 epitope tag for immuno-detection was ectopically expressed in the 3D7 parasite line under the AMA1 promoter (Figure 5A). To test the invasion capability of phosphorylation defective mutants, a S610A mutation (AMA1-S610A-TY1) or mutations of all six predicted phosphorylation sites in the AMA1 tail to alanines (AMA1-PM-TY1) were introduced by site-directed mutagenesis (Figure 5B). Importantly, all the AMA1-TY1 proteins, wild type and mutants, were derived from the W2mef parasite strain. In this strain the AMA1 protein bears crucial amino acid changes that makes it resistant to the invasion blocking effects of the R1 peptide that binds to the 3D7 AMA1 protein [28]. This strain specific binding blocks RBC invasion by 3D7 parasites though it does not prevent initial interaction steps between the merozoite and RBC [18]. All chimeric proteins were correctly expressed as TY1 fusion proteins (Figure 5C, left panel). Proteolytic cleavage of the HA-fusion was indistinguishable from the endogenous protein as shown with the 3D7 specific monoclonal AMA1 antibody 1F9 [29] (Figure 5C, right panel). As previously reported, over-expression of AMA1-WT-TY1 (derived from W2mef) functionally complements the endogenous AMA1 (∼78.5% (+/− 5.1% s.d.) invasion) while both mutants AMA1-S610A-TY1 and AMA1-PM-TY1 revealed a drastically decreased invasion capability in the presence of R1 peptide (∼21.2% (+/−5.9% s.d.) and ∼20.3% (+/− 5.2% s.d.) invasion, respectively) (Figure 5D). In comparison, invasion is blocked up to ∼96% (+/− 1.3% s.d.) in the parental 3D7 parasite line with the R1 peptide. This comparable failure of the PM and the S610A mutant to complement AMA1 function demonstrates an important role for AMA1 residue S610 and, together with the above *in vitro* data, strongly implicates PKA-mediated phosphorylation of S610 as a vital step in promoting efficient invasion. Direct comparison of the temporal aspect of invasion blockade induced by either R1 peptide binding or S610A mutation using video-microscopy revealed no apparent differences. Both parasites are arrested after the re-orientation of the merozoite on the surface of the host cell (Video S1, Figure S2). In summary, this data demonstrates that the cytoplasmic tail of AMA1 is phosphorylated by parasite-encoded PKA, and that this is crucial to AMA1 function during the invasion. The vital role of S610 was further investigated by targeting two other single amino acids that are predicted and could be putatively involved in phosphorylation of AMA1: Y576 and Y585. The substitution of these amino acids with alanine and its subsequent ectopic expression (AMA1-Y576A-TY1 and AMA1-Y585A-TY1) did not impair their function in complementation assays (Figure 5D). Additionally, we have tried to mimic the phosphorylation state of AMA1 by expressing AMA1-S610E-TY1 and AMA1-S610D-TY1 mutants in the presence of R1 peptide, however they failed to complement the invasion efficiency of the AMA1-WT-TY1 allele and performed little better than the non-phosphorylatable S610A allele (Figure 5D). This could be because the negatively charged amino acids fail to fully substitute the biological activity of a phosphate group or because they cannot lose their charge through the action of phosphatases. The latter seems a distinct possibility since the 2D pattern of AMA1 spots in ring-stage parasites is greatly simplified compared to merozoites suggesting the activity of phosphatases.

## Discussion

Merozoite invasion of RBCs is a rapid yet complex multi-step process. It can be broadly separated into a ∼11 sec pre-invasion stage which is characterised by long-distance interactions and extensive deformation of the host cell and a ∼17 second invasion step where the parasite has formed a moving tight junction [30]. The pre-invasion stage is particularly poorly understood both in terms of the receptor-ligand interactions involved and in the communication events that link the subsequent stages of invasion. However, it appears likely that during the pre-invasion stage a signalling cascade occurs to promote subsequent invasion steps, such as apical organelle secretion and activation of the actin-myosin motor. Most notably in this regard calcium dependent kinase 1 (CDPK1) has been implicated to be involved in phosphorylating motor components [31].

In the present work, we have shown that the major transmembrane protein known to be present on the surface of free merozoites AMA1, appears to be phosphorylated at a specific residue, S610, in its cytoplasmic tail. We show that this occurs both in *in vitro* assays using recombinant AMA1 with parasite extracts and also *in vivo* in whole parasites where S610 appears to be a dominant site of phosphorylation. Given that we observed 3 phosphorylated isoforms of AMA1 it remains possible that another site(s) of the AMA1 tail is/are also phosphorylated. However, if this is the case, these sites must be subsequent to and dependent of serine 610 phosphorylation as all isoforms are absent when this residue is mutated. Several lines of evidence indicate that the enzyme responsible for this event is parasite-encoded *Pf*PKA, an enzyme previously unknown to be involved in invasion. Firstly, *in vitro* S610 is phosphorylated strongly in a cAMP dependent manner and *Pf*PKA is the only known cAMP dependent kinase expressed in the blood stages of *P. falciparum*. In fact, *Pf*PKA transcription is co-regulated with that of AMA1 late in the blood-stage cycle and S610 is strongly predicted by publicly available software to constitute a PKA site. Secondly, two different PKA inhibitors H89 and KT5720 block this phosphorylation *in vitro*. Thirdly, we show that immuno-precipitated *Pf*PKA phosphorylates the tail of AMA1 *in vitro.* Finally we use a complementation approach to show that S610 is important for efficient red blood cell invasion. In this experiment, mutant parasites that express an AMA1 with an alanine in this position are unable to efficiently invade host cells. Taken together, we conclude that *Pf*PKA mediated phosphorylation of AMA1 at S610 is vital to its functioning and hence to the invasion process.

So what is the role of this process in invasion and what can we learn from host cell entry at other parasite stages? With respect to the latter, *Pf*PKA is also expressed in the hepatocyte invasive sporozoite stages and indeed PKA has been implicated in invasion via genetic deletion of *P. berghei* adenylyl cyclase alpha (ACα; [32]). ACα is responsible for rapidly generating cAMP from ATP in response to a stimulus, in this case uracil derivates [32]. The membrane associated ACα is not expressed in blood-stages however another adenylyl cyclase, ACβ, is expressed during the blood-stages [33]. In fact, the gene encoding ACβ is co-transcribed with genes encoding PKA and AMA1 late in the blood-stage cycle. Hence, ACβ is a strong candidate to produce the cAMP required to activate PKA during invasion. Experimental confirmation of this is required but if true the identification of the activation signals of ACβ should shed considerable light on the primary trigger for merozoite invasion of red blood cells.

It will be interesting to discover how the phosphorylation of AMA1 renders the merozoites competent to invade. Phosphorylation does not appear to influence AMA1 trafficking since expression of a tail deletion mutant in transgenic parasites does not appear to effect localisation [18]. Previous experiments have indicated that the interaction of AMA1 with RON proteins is essential for tight junction formation [14], [34]. Does phosphorylation of the AMA1 interfere with RON/AMA1 complex formation? This appears unlikely as AMA1 without the cytoplasmic tail (like wild type AMA1) remains capable of interacting with RON4 [18]. It remains to be determined whether the phosphorylation of the AMA1 tail by PKA has a direct effect on an AMA1 binding function or whether this event is a key step in signal transduction pathway. It is interesting to note that the AMA1-S610A-TY1 mutant invades at 20% of AMA1-WT-TY1 levels in the presence of R1 peptide whereas in the absence of a complementing W2mef protein the 3D7 parasites only invade with ∼4% efficiency. This indicates that there is not an absolute need for S610 phosphorylation for every successful invasion or that there is some cross-talk between the native 3D7 protein whose tail can presumably still be phosphorylated and the W2mef AMA1-S610A-TY1 mutant that cannot be phosphorylated but whose ectodomains probably remain functional.

It is interesting to consider the likelihood of a link between cAMP signalling and calcium signalling during merozoite invasion. Both the Ono et al. study in sporozoites [32] and earlier work in *P. falciparum* blood-stages [20] have demonstrated an inter-relationship between these two pathways and indeed in higher order eukaryotes this relationship is now well established (reviewed in [35]). How these are involved in invasion by merozoites and sporozoites is unclear although it appears likely that cAMP signalling operates upstream of intracellular calcium release [20], [32].

In summary, this study opens up a new area of investigation for those interested in understanding *P. falciparum* merozoite invasion. Knowledge of the molecular events that trigger PKA signalling as well as those that follow as a consequence of AMA1 phosphorylation will be essential to a full understanding of merozoite invasion.

## Materials and Methods

### Ethics statement

The culture of malaria parasites using donated blood and serum from the Australian Red Cross Society has been approved by The Walter and Eliza Hall Institute Human Research Ethics Committee and by the Bernhard Nocht Institute. The use of this material follows long-standing protocols and has not been associated with any adverse or other unforeseen events and no data of relevance to specific patients has been generated. A Supply Agreement has been executed between the Australian Red Cross Society and the Walter and Eliza Hall Institute and between the Australian Red Cross Society and the Burnet Institute covering the provision of blood and blood products for non-clinical use.

### *In vitro* phosphorylation assay

Magnetic cell-sorted *P. falciparum* 3D7 parasites were cultured in the absence of RBCs until about half of the schizonts had ruptured to release their merozoites. A schizont enriched fraction was prepared by centrifuging the parasite culture at 1500×g for 5 min to pellet the schizonts. To harvest the merozoites, the supernatant was then spun at 3000×g for 15 mins and enrichment process was checked by microscopy of Giemsa-stained smears of the schizonts and merozoites. Schizonts were then released from host cells by saponin lysis. To make parasite lysates with kinase activity, the parasite pellets were lysed in 10 volumes of ice cold buffer B (50 mM β-glycerophosphate pH 7.3, 1% Triton X-100, 1 mM DTT, Complete Protease Inhibitor Cocktail [Roche] and phosphatase inhibitors, 50 mM Na_3_VO_4_ and 50 mM NaF,) and incubated on a rotating wheel overnight at 4°C. Control extracts were made from similar amounts of whole uninfected red blood cells under identical conditions. To clear the lysates they were centrifuged at 16000×g and the protein concentration of the supernatant was determined by BCA protein assay (Pierce). 3 µg total protein lysate (or 1 µL of immuno-precipitated *Pf*PKA-HA-sepharose) was mixed with ∼10 µg of the GST or GST-AMA1-tail fusion proteins immobilized on glutathione-sepharose beads. To the beads buffer B containing different compounds was added to a final volume of 100 µL. The compounds used were 1.5 mM EGTA/1 mM EDTA, 2 mM CaCl_2_, 1 µM cAMP, 10 µM KT 5720, 50 µM H89 and 1% methanol for a control. The concentration of stock solutions, solvents and replicate numbers are indicated in the supplementary data (Text S1). The reaction was initiated by the addition of 3 µL ATP mixture composed of 100 µL 1 M MgCl_2_ and 150 µCi ^32^[P]ATP (10 µCi/mL). After 30 minutes at 30°C, the immobilised fusion proteins were pelleted and washed in buffer B. The AMA1-tail was removed from the beads as whole GST-AMA1-tail fusion protein with 10 mM glutathione in PBS or the tail only was cleaved from the GST with thrombin. Eluted proteins were immediately suspended in SDS gel sample buffer and were boiled for 5 min, resolved on a 4–12% gradient SDS-PAGE gel, blotted onto a PVDF membrane. Imaging of ^32^[P]-labelled protein bands was achieved by direct autoradiography (1–2 day exposure) of dry blots using FUJIFILM BAS-TR2040 tritium imaging plates and a FLA-3000 luminometric detection system.

### *Pf*PKA-HA immunoprecipitation

PKA-HA was immunoprecipitated using anti-HA antibodies (5 µg/mL) and protein-G-sepharose (Invitrogen). 20 mL of a 10% synchronized schizont culture (PfPKA-HA expressing and wild type parasite strain) were saponin lysed. The resulting parasite pellet was washed in icecold PBS and hypotonically lysed in 10 volumes of ice-cold buffer B without detergent by several passages through a needle and 2 hours incubation on a rotating wheel at 4°C. The membrane fraction was separated from the soluble proteins by centrifugation at 13.000×g for 30 minutes. The resulting supernatant was precleared with protein-G-sepharose beads. The precleared lysate was incubated with anti-HA antibodies with a final concentration of 5 µg/mL for 3 hours. 20 µL of protein-G-sepharose was used to precipitate the PKA-HA antibody complexes. It was washed 5x with 10 volumes of cold PBS and subsequently stored in buffer B at −80°C until used. For each experiment, 3 µL of PKA-HA coupled beads were used.

### Erythrocyte invasion assays

Parasite erythrocyte invasion assays were performed using 3D7 and transgenic parasites AMA1-WT-TY1, AMA1-S610A-TY1 and AMA1-PM-TY1 (3D7 background). Parasitemia of sorbitol synchronized parasite culture was measured using the FACS. For the experiment a parasite culture with 0.5–1% parasitemia of late trophozoites (4% hematocrit) was incubated in a 96-well Plate (100 µL per well) under standard culturing conditions for 48 hours to allow re-infection in the presence or absence (control) of 100 mg/mL R1. After reinvasion occurred, parasites were stained with 1 mg/mL ethidium bromide for 30 minutes at 37°C, washed three times with media and then counted using the FACS. Assays were performed in triplicates on three independent occasions.

### Metabolic labelling of phosphorylated *P. falciparum* protein

Metabolic labelling of phosphorylated parasite protein was achieved by incubating ∼1×10^10^ sorbitol-synchronized *P. falciparum* 3D7 parasites with 100 µCi/mL of ^32^[P]-monosodium phosphate (Perkin-Elmer) in 50 mL phosphate-free RPMI medium (Gibco) supplemented with 25 mM HEPES (pH 7.2), 0.5% (w/v) Albumax, 0.4% (w/v) glucose, 0.2% (w/v) Na_2_HCO_3_ at 37°C overnight. Late blood-stage parasites were released from host cells by saponin lysis, extensively washed in TBS, and parasite pellets extracted in 10 volumes of HNET (25 mM HEPES, pH 7.4, 150 mM NaCl, 5 mM EDTA, 1% Triton X-100) for one hour with vortexing. Detergent-soluble schizont extract was clarified by ultracentrifugation at 75,000×*g* for 30 mins at 4°C. All procedures were carried out in the presence of protease and phosphatase inhibitors on ice, unless otherwise stated. ^32^[P]-labelled parasite extracts were snap-frozen in liquid nitrogen and stored at -80°C until required.

### 2D gel electrophoresis (2DE)

Two-dimensional gel electrophoresis was performed using conditions required for optimal extraction and separation of *P. falciparum*-infected erythrocyte proteins [36]. Frozen parasite extracts were processed using 2-D Clean-Up Kit (GE Healthcare). The resulting precipitates (∼100 mg protein) were redissolved in 300 µL 2-DE sample buffer (7 M urea, 2 M thiourea, 2% ASB-14, 1% DTT, 1% ampholytes), loaded onto 13 cm *pI* 4–7 IPG strips by passive re-hydration for 12 hours, and focussed using a fast voltage gradient (8000V max, 24,000 Vh) at 15°C, using an Ettan IPGphor 3 system (GE Healthcare). The second dimension was carried out on 7.5% polyacrylamide gels using a Hoefer SE 600 system (GE Healthcare) at 75V overnight.

### Protein staining, autoradiography and Western analyses of 2DE blots

2-DE gels were electrophoretically transferred onto Immobilon-P^SQ^ PVDF membranes (Millipore) in Towbin's transfer buffer containing 20% methanol and 0.01% SDS. Complete transfer of total protein was confirmed using Deep Purple protein stain (GE Healthcare). Imaging of ^32^[P]-labelled parasite phospho-protein spots was achieved by direct autoradiography of dry blots using FUJIFILM BAS-TR2040 tritium imaging plates and a FLA-3000 luminometric detection system (14 day exposure). 2DE Western blot analyses of protein extracts of 3D7 parasites expressing wild type and/or transgenic AMA1-WT-TY1 or AMA1-S610A-TY1 was carried out as detailed in the figure legend.

### Nucleic acids and DNA constructs

*The ama1* gene was either amplified from 3D7 or W2mef *P. falciparum* gDNA (Table S1). For the generation of GST fusion proteins the DNA sequence of the *Pf*AMA1 C-terminal tail was cloned into *Bam*HI and *Eco*RI restriction sites of the bacterial expression vector pGEX-4T-3 (Pharmacia Biotech). This construct produces fusion proteins of *Pf*AMA1 tail C-terminally bound to glutathione S-transferase (GST). Different mutants of the GST-*Pf*AMA1 tail fusion protein were achieved by using a site-directed mutagenesis kit (Stratagene) and sequences were confirmed by sequencing (Table S1).

For transfecting 3D7 parasites *ama1* was cloned into the *Kpn*I and *Avr*II restriction sites of the pARL-AMA1-GFP Vector and sequences were confirmed by sequencing. To ensure correct timing of transcription, expression of the AMA1 transgenes were controlled by the AMA1 promoter. *In vitro* mutagenesis of *ama1* was achieved by using a two-step primer directed PCR mutagenesis method (Table S1) with proof reading Vent polymerase (NEB).

### Parasites strains and transfection

*P. falciparum* asexual stages were cultured in human 0^+^ erythrocytes according to standard procedures. W2mef is derived from the Indochina III/CDC strain. 3D7 parasites were transfected with 100 µg of purified plasmid DNA. Positive selection for transfectants was achieved using 10 nM WR99210, an antifolate that selects for the presence of the human *dhfr* gene.

For further and more detailed information see Text S1.

## Supporting Information

Figure S1 (A) Western Blot of ectopically expressed PfPKA-HA using anti-HA antibodies. Whereas in wild type parasite material (WT) no fusion protein was detectable two protein bands with the predicted size of approximately 43 kDa were visualized in the transgenic parasite line 3D7PfPKA-HA. (B) Immuno-fluorescence images of PfPKA-HA expressing parasites 3D7PfPKA-HA revealed cytosolic distribution (green) using anti-HA antibodies. Blue: DAPI stained nucleus.
(0.14 MB DOC)

Figure S2 M1 video file depicted in time-lapse micrographs. After schizont rupture (t = 20s) free merozoites attack an erythrocyte (black arrows (t = 28s). Around 40 seconds after primary contact (t = 63s) the erythrocyte looses its shape - culminating in a spiked round structure with at least three merozoites apically attached to it (red arrows).
(0.22 MB DOC)

Table S1 Oligonucleotides used in this study.
(0.05 MB DOC)

Text S1 Supplemental materials and methods.
(0.06 MB DOC)

Video S1 Video-microscopy of attempted invasion event of AMA1-S610A-TY1 expressing parasites in the presence of the R1 peptide. Although the merozoites are able to attack and induce echinocytosis in the erythrocyte, invasion is blocked.
(1.3 MB MPG)

## References

[1] Snow RW, Guerra CA, Noor AM, Myint HY, Hay SI (2005). The global distribution of clinical episodes of *Plasmodium falciparum* malaria.. Nature.

[2] Cowman AF, Crabb BS (2006). Invasion of red blood cells by malaria parasites.. Cell.

[3] Bannister L, Mitchell G (2003). The ins, outs and roundabouts of malaria.. Trends Parasitol.

[4] Healer J, Crawford S, Ralph S, McFadden G, Cowman AF (2002). Independent translocation of two micronemal proteins in developing *Plasmodium falciparum* merozoites.. Infect Immun.

[5] Hodder A, Crewther P, Matthew M, Reid G, Moritz R (1996). The disulfide bond structure of *Plasmodium* apical membrane antigen-1.. J Biol Chem.

[6] Peterson MG, Marshall VM, Smythe JA, Crewther PE, Lew A (1989). Integral membrane protein located in the apical complex of *Plasmodium falciparum*.. Mol Cell Biol.

[7] Narum DL, Thomas AW (1994). Differential localization of full-length and processed forms of PF83/AMA-1 an apical membrane antigen of *Plasmodium falciparum* merozoites.. Mol Biochem Parasitol.

[8] Harris PK, Yeoh S, Dluzewski AR, O'Donnell RA, Withers-Martinez C (2005). Molecular identification of a malaria merozoite surface sheddase.. PLoS Pathog.

[9] Howell SA, Well I, Fleck SL, Kettleborough C, Collins CR (2003). A single malaria merozoite serine protease mediates shedding of multiple surface proteins by juxtamembrane cleavage.. J Biol Chem.

[10] Mital J, Meissner M, Soldati D, Ward GE (2005). Conditional expression of *Toxoplasma gondii* apical membrane antigen-1 (TgAMA1) demonstrates that TgAMA1 plays a critical role in host cell invasion.. Mol Biol Cell.

[11] Mitchell GH, Thomas AW, Margos G, Dluzewski AR, Bannister LH (2004). Apical membrane antigen 1, a major malaria vaccine candidate, mediates the close attachment of invasive merozoites to host red blood cells.. Infect Immun.

[12] Urquiza M, Suarez JE, Cardenas C, Lopez R, Puentes A (2000). *Plasmodium falciparum* AMA-1 erythrocyte binding peptides implicate AMA-1 as erythrocyte binding protein.. Vaccine.

[13] Bai T, Becker M, Gupta A, Strike P, Murphy VJ (2005). Structure of AMA1 from *Plasmodium falciparum* reveals a clustering of polymorphisms that surround a conserved hydrophobic pocket.. Proc Natl Acad Sci U S A.

[14] Collins CR, Withers-Martinez C, Hackett F, Blackman MJ (2009). An inhibitory antibody blocks interactions between components of the malarial invasion machinery.. PLoS Pathog.

[15] Kocken CH, van der Wel AM, Dubbeld MA, Narum DL, van de Rijke FM (1998). Precise timing of expression of a Plasmodium falciparum-derived transgene in *Plasmodium berghei* is a critical determinant of subsequent subcellular localization.. J Biol Chem.

[16] Alexander DL, Arastu-Kapur S, Dubremetz JF, Boothroyd JC (2006). Plasmodium falciparum AMA1 binds a rhoptry neck protein homologous to TgRON4, a component of the moving junction in Toxoplasma gondii.. Eukaryot Cell.

[17] Alexander DL, Mital J, Ward GE, Bradley P, Boothroyd JC (2005). Identification of the moving junction complex of *Toxoplasma gondii*: a collaboration between distinct secretory organelles.. PLoS Pathog.

[18] Treeck M, Zacherl S, Herrmann S, Cabrera A, Kono M (2009). Functional analysis of the leading malaria vaccine candidate AMA-1 reveals an essential role for the cytoplasmic domain in the invasion process.. PLoS Pathog.

[19] Nagamune K, Moreno SN, Chini EN, Sibley LD (2008). Calcium regulation and signaling in apicomplexan parasites.. Subcell Biochem.

[20] Beraldo FH, Almeida FM, da Silva AM, Garcia CR (2005). Cyclic AMP and calcium interplay as second messengers in melatonin-dependent regulation of *Plasmodium falciparum* cell cycle.. J Cell Biol.

[21] Syin C, Parzy D, Traincard F, Boccaccio I, Joshi MB (2001). The H89 cAMP-dependent protein kinase inhibitor blocks *Plasmodium falciparum* development in infected erythrocytes.. Eur J Biochem.

[22] Bozdech Z, Zhu J, Joachimiak MP, Cohen FE, Pulliam B (2003). Expression profiling of the schizont and trophozoite stages of *Plasmodium falciparum* with a long-oligonucleotide microarray.. Genome Biol.

[23] Ward P, Equinet L, Packer J, Doerig C (2004). Protein kinases of the human malaria parasite *Plasmodium falciparum*: the kinome of a divergent eukaryote.. BMC Genomics.

[24] Murray AJ (2008). Pharmacological PKA inhibition: all may not be what it seems.. Sci Signal.

[25] Steinberg RA, Cauthron RD, Symcox MM, Shuntoh H (1993). Autoactivation of catalytic (C alpha) subunit of cyclic AMP-dependent protein kinase by phosphorylation of threonine 197.. Mol Cell Biol.

[26] Tang Y, McLeod M (2004). In vivo activation of protein kinase A in *Schizosaccharomyces pombe* requires threonine phosphorylation at its activation loop and is dependent on PDK1.. Genetics.

[27] Healer J, Triglia T, Hodder AN, Gemmill AW, Cowman AF (2005). Functional analysis of *Plasmodium falciparum* apical membrane antigen 1 utilizing interspecies domains.. Infect Immun.

[28] Harris KS, Casey JL, Coley AM, Masciantonio R, Sabo JK (2005). Binding hot spot for invasion inhibitory molecules on *Plasmodium falciparum* apical membrane antigen 1.. Infect Immun.

[29] Coley AM, Parisi K, Masciantonio R, Hoeck J, Casey JL (2006). The most polymorphic residue on *Plasmodium falciparum* apical membrane antigen 1 determines binding of an invasion-inhibitory antibody.. Infect Immun.

[30] Gilson PR, Crabb BS (2009). Morphology and kinetics of the three distinct phases of red blood cell invasion by *Plasmodium falciparum* merozoites.. Int J Parasitol.

[31] Green JL, Rees-Channer RR, Howell SA, Martin SR, Knuepfer E (2008). The motor complex of P*lasmodium falciparum*: phosphorylation by a calcium-dependent protein kinase.. J Biol Chem.

[32] Ono T, Cabrita-Santos L, Leitao R, Bettiol E, Purcell LA (2008). Adenylyl cyclase alpha and cAMP signaling mediate *Plasmodium* sporozoite apical regulated exocytosis and hepatocyte infection.. PLoS Pathog.

[33] Baker DA (2004). Adenylyl and guanylyl cyclases from the malaria parasite Plasmodium falciparum.. IUBMB Life.

[34] Besteiro S, Michelin A, Poncet J, Dubremetz JF, Lebrun M (2009). Export of a *Toxoplasma gondii* rhoptry neck protein complex at the host cell membrane to form the moving junction during invasion.. PLoS Pathog.

[35] Borodinsky LN, Spitzer NC Second messenger pas de deux: the coordinated dance between calcium and cAMP.. Sci STKE.

[36] Rabilloud T, Blisnick T, Heller M, Luche S, Aebersold R (1999). Analysis of membrane proteins by two-dimensional electrophoresis: comparison of the proteins extracted from normal or *Plasmodium falciparum*-infected erythrocyte ghosts.. Electrophoresis.

