# Supplementary figures and images for: Protein Kinase A Dependent Phosphorylation of Apical Membrane Antigen 1 Plays an Important Role in Erythrocyte Invasion by the Malaria Parasite
Source: PLoS Pathog. 2010 Jun 3;6(6):e1000941. doi: 10.1371/journal.ppat.1000941 (PMC2880582; doi:10.1371/journal.ppat.1000941)

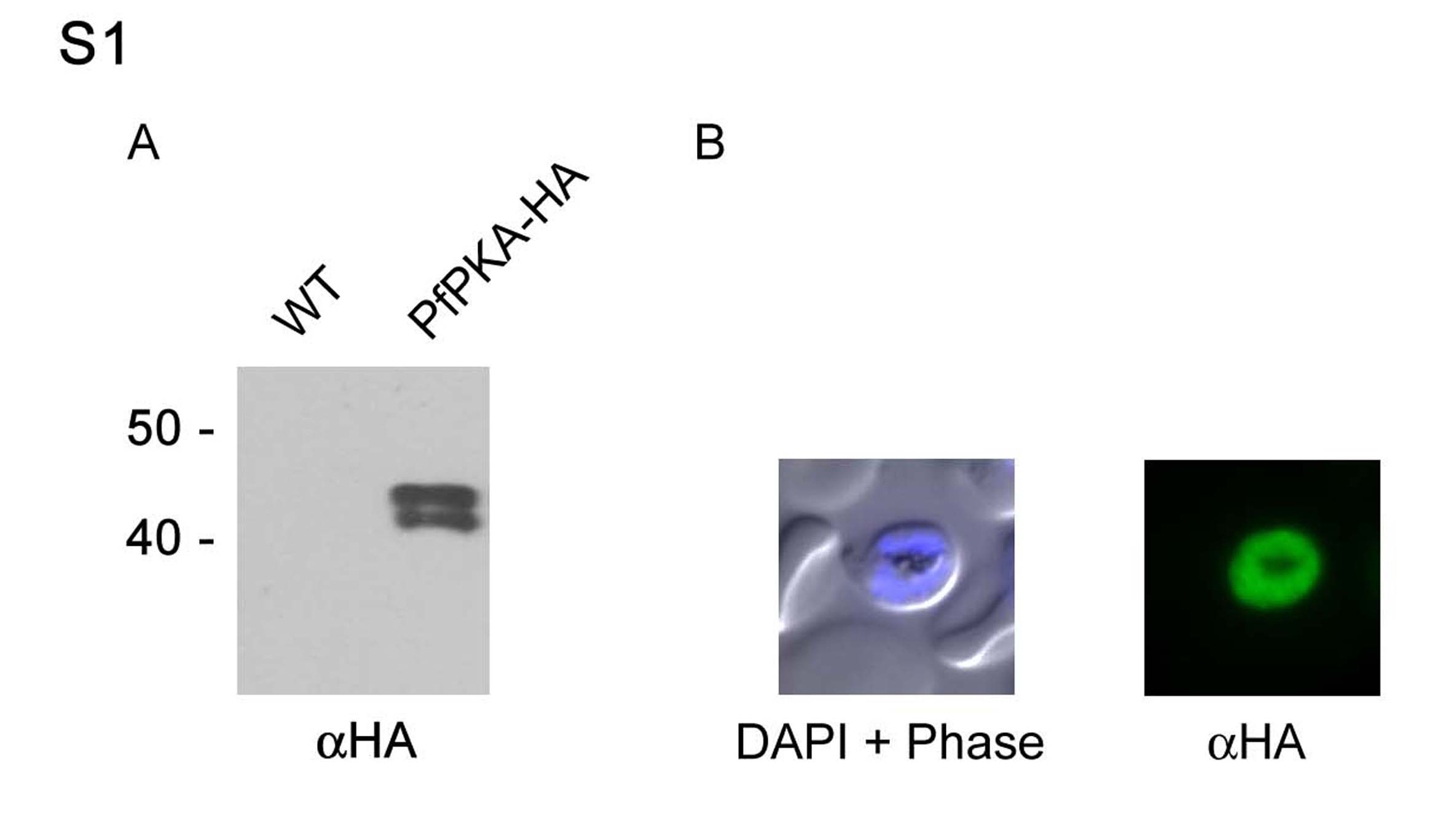

Supplement: Figure S1 — (A) Western Blot of ectopically expressed PfPKA-HA using anti-HA antibodies. Whereas in wild type parasite material (WT) no fusion protein was detectable two protein bands with the predicted size of approximately 43 kDa were visualized in the transgenic parasite line 3D7PfPKA-HA. (B) Immuno-fluorescence images of PfPKA-HA expressing parasites 3D7PfPKA-HA revealed cytosolic distribution (green) using anti-HA antibodies. Blue: DAPI stained nucleus. (0.14 MB DOC) [file ppat.1000941.s001.doc]

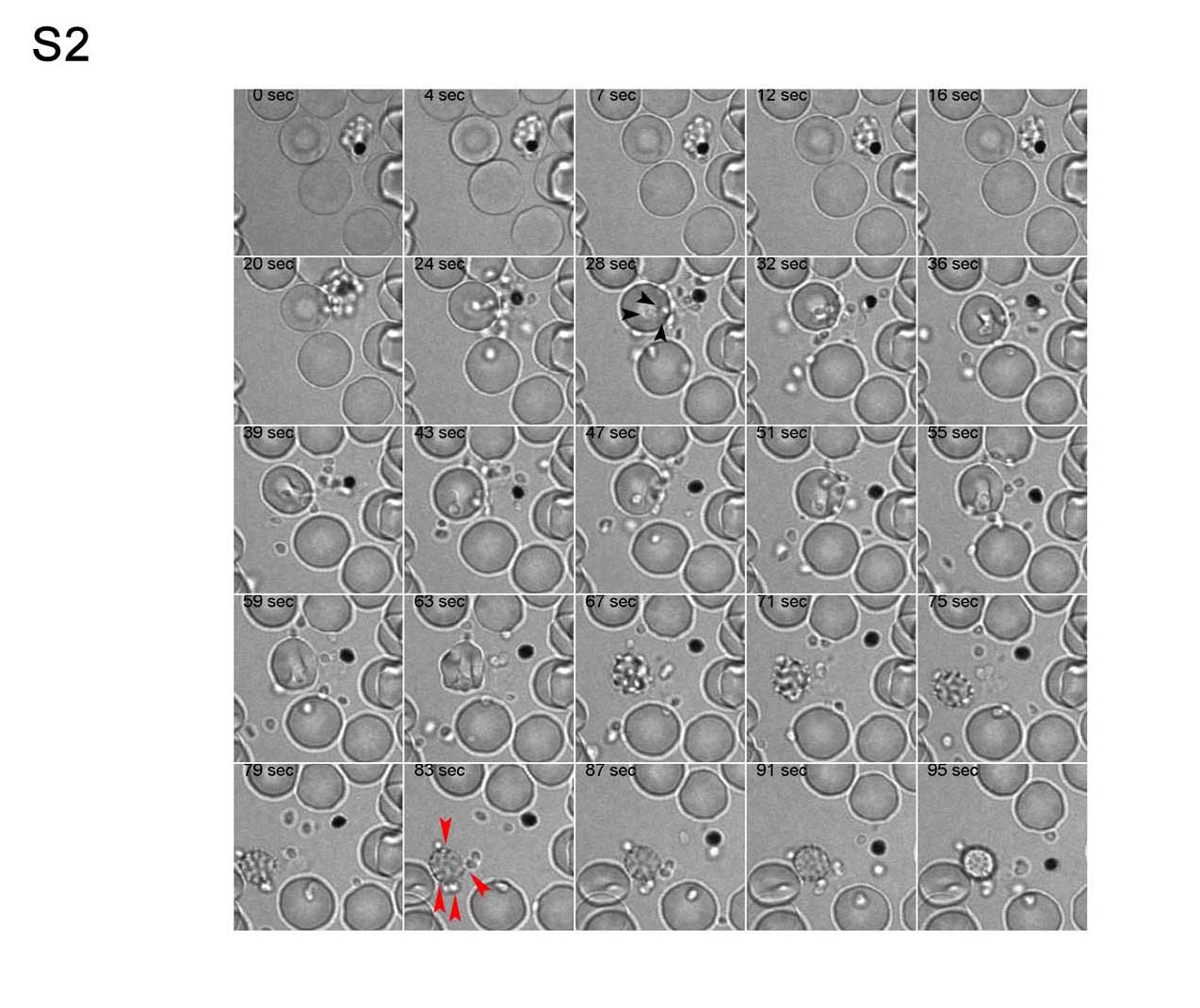

Supplement: Figure S2 — M1 video file depicted in time-lapse micrographs. After schizont rupture (t = 20s) free merozoites attack an erythrocyte (black arrows (t = 28s). Around 40 seconds after primary contact (t = 63s) the erythrocyte looses its shape - culminating in a spiked round structure with at least three merozoites apically attached to it (red arrows). (0.22 MB DOC) [file ppat.1000941.s002.doc]
