# Supplementary material for: Protein Kinase A Dependent Phosphorylation of Apical Membrane Antigen 1 Plays an Important Role in Erythrocyte Invasion by the Malaria Parasite
Source: PLoS Pathog. 2010 Jun 3;6(6):e1000941. doi: 10.1371/journal.ppat.1000941 (PMC2880582; doi:10.1371/journal.ppat.1000941)
Supplement: Table S1 — Oligonucleotides used in this study. (0.05 MB DOC) [file ppat.1000941.s003.doc]

**Table S1:** Oligonucleotides used in this study

| **Primer** | **Sequence** |
| --- | --- |
| AMA1 GST-CTer-F | TAATGGTTGGATCCTATAAAAGAAAAGGAAATGCTGAAAAATATG |
| AMA1 GST-CTer-R | ATAGTTGAATTCTTAATAGTATGGTTTTTCCATCAGAACTGG |
| F-AMA1CTer-S610stop | GGGGGGAAGAAAAAAGAGCATGACATACAACACCAGTTCTG |
| R-AMA1CTer-S610stop | CAGAACTGGTGTTGTATGTCATGCTCTTTTTTCTTCCCCCC |
| F-AMA1CTer-S601A | GAAATGTTAGATCCTGAGGCAGCTTTTTGGGGGGAAGAAAAAAGAG |
| R-AMA1CTer-S601A | CTCTTTTTTCTTCCCCCCAAAAAGCTGCCTCAGGATCTAACATTTC |
| F-AMA1CTer-S610A | GGGGGGAAGAAAAAAGAGCAGCACATACAACACCAGTTCTG |
| R-AMA1CTer-S610A | CAGAACTGGTGTTGTATGTGCTGCTCTTTTTTCTTCCCCCC |
| F-AMA1CTer-T612A | GGAAGAAAAAAGAGCATCACATGCAACACCAGTTCTGATGGAAAAACC |
| R-AMA1CTer-T612A | GGTTTTTCCATCAGAACTGGTGTTGCATGTGATGCTCTTTTTTCTTCC |
| F-AMA1CTer-T613A | GGAAGAAAAAAGAGCATCACATACAGCACCAGTTCTGATGGAAAAACC |
| R-AMA1CTer-T613A | GGTTTTTCCATCAGAACTGGTGCTGTATGTGATGCTCTTTTTTCTTCC |
| F-AMA1CTer-Y621A | CCAGTTCTGATGGAAAAACCAGCCTATTAAGAATTCCCGGGTCG |
| R-AMA1CTer-Y621A | CGACCCGGGAATTCTTAATAGGCTGGTTTTTCCATCAGAACTGG |
| F-AMA1CTer-Y622A | GTTCTGATGGAAAAACCATACGCTTAAGAATTCCCGGGTCGAC |
| R-AMA1CTer-Y622A | GTCGACCCGGGAATTCTTAAGCGTATGGTTTTTCCATCAGAAC |
| AMA1-S | GCGCGGTACCATGAGAAAATTATACTGCG |
| Y576-S | AAAAGAAAAGGAAATGCTGAAAAAGCAGATAAAATGGATGAACCACAAGATTATGGGA |
| Y576-AS | CCCATAATCTTGTGGTTCATCCATTTTATCTGCTTTTTCAGCATTTCCTTTTCTTTT |
| Y585-S | GATAAAATGGATGAACCACAACATGCTGGGAAATCAAATGCAGCGAATG |
| Y585-AS | CATTCGCTGCATTTGATTTCCCAGCATGTTGTGGTTCATCCATTTTATC |
| S610A-AS | GCGCCCTAGGATAGTATGGTTTTTCCATCAGAACTGGTGTTGTATGTGCTGCTCTTTTTTCTTCCCCCC |
| S610E-AS | GCGCCCTAGGATAGTATGGTTTTTCCATCAGAACTGGTGTTGTATGTTCTGCTCTTTTTTCTTCCCCCC |
| S610D-AS | GCGCCCTAGGATAGTATGGTTTTTCCATCAGAACTGGTGTTGTATGGTCTGCTCTTTTTTCTTCCCCCC |
| PKAc-S | GCGCGGTACCATGCAGTTTATTAAAAATTTGC |
| PKAc-AS | GCGCCCTAGGCCAATCATAAAATGGATCATTTTCATTTG |
