# Supplementary material for: Protein Kinase A Dependent Phosphorylation of Apical Membrane Antigen 1 Plays an Important Role in Erythrocyte Invasion by the Malaria Parasite
Source: PLoS Pathog. 2010 Jun 3;6(6):e1000941. doi: 10.1371/journal.ppat.1000941 (PMC2880582; doi:10.1371/journal.ppat.1000941)
Supplement: Text S1 — Supplemental materials and methods. (0.06 MB DOC) [file ppat.1000941.s004.doc]

**Supplemental Materials and Methods:**

#### Reagents

1 μM cAMP (Sigma-Aldrich), 2 mM CaCl_2_ (AnalaR), 1.5 mM EGTA (Sigma-Aldrich), 1.0 mM EDTA (Univar), 50 μM H89 (Sigma-Aldrich), 10 μM KT5720 (Sigma-Aldrich), 100 mg/mL R1 peptide (Genscript), Complete protease inhibitor cocktail tablets (Roche), Phosphatase inhibitor cocktail 1 (Invitrogen).

| compound | Stock concentration | Dissolved in | Final concentration |
| --- | --- | --- | --- |
| cAMP | 10 mM | H_2_O | 1 μM |
| CaCl_2_ | 200 mM | H_2_O | 2 mM |
| EGTA | 100 mM | H_2_O | 1.5 mM |
| EDTA | 100 mM | H_2_O | 1.0 mM |
| H89 | 10 mM | H_2_O | 50 μM |
| KT5720 | 1 mM | methanol | 10 μM |

**Number of experimental replicates in *in vitro* phosphorylation assays:**

+ EGTA/EDTA: 2x

+ CaCl_2_: 2x

+ cAMP: 4x

+ H89: 3x

+ KT5720: 2x

+ cAMP + H89: 1x

+ cAMP + KT5720: 2x

+ methanol: 2x

**Expression and Purification of GST fusion proteins.**

GST fusion plasmids and the empty pGEX-4T-3 vector (Pharmacia Biotech) were transformed into *Escherichia coli* strain BL21. Bacteria were grown overnight, 6 mL were transferred into 600 mL of fresh LB, and incubated at 37°C until an OD_600_ 0.5-0.6 was reached. Expression was induced by addition of 300 μM Isopropyl-beta-D-thiogalactopyranoside and incubation at 37°C for 1.5 hours. The bacterial pellet was collected by centrifugation and resuspended with PBS. Cells were lysed by sonication and the extract was cleared by centrifugation. To prepare GST-fusion protein-coated beads, the supernatant was bound to glutathione-sepharose 4B beads (Amersham Pharmacia). The desired proteins immobilized on beads were resuspended in buffer B (50 mM β-glycerophosphate pH 7.3, 1 mM DTT, protease and phosphatase inhibitors).

## **Antisera and Western Blots**

Antibodies used in Western Blots were rabbit polyclonal anti-AMA1 antibodies detecting specifically the C-terminal tail of AMA1 (1:500), mouse monoclonal anti-TY1 antibodies (1:1000, Diagenode). Mouse monoclonal 1F9 anti-AMA1 (31) was used 1:2000 and rat anti-HA (Roche) was used 1:1000. Antibodies were diluted in phosphate-buffered saline (PBS) with 3% w/v skim milk or 3% BSA (for immuno-fluorescence assays). Immuno-blots were performed using standard procedures and bound antibody probes were detected with LiCor Odyssey infrared imager or developed by chemiluminescence using ECL (Amersham International). Secondary antibodies were IRDye®700DX and IRDye®800CW conjugated goat anti-rabbit IgG (Rockland Immunochemicals), sheep anti-mouse IgG horseradish peroxidase (Roche), all used 1:5000, HRP conjugated anti-rat IgG (Cell Signalling 1:2000) or Alexa488 conjugated anti-rat-IgG (Molecular Probes, 1:2000).

**Immunofluorescence assays**

*Pf*PKA-HA-transfected parasites smears of synchronized late schizont stages were fixed in ice cold methanol for 4 minutes rehydrated and subsequently blocked with 3% BSA. *Pf*PKA-HA was detected using HA specific antibodies. Nuclei were stained with DAPI (1:2000). Images were taken using a Zeiss Axioscope with 100x magnification. ImageJ was used as image software .

**Life video microscopy**

Life video microscopy was performed using a Zeiss LSM with a temperature and CO_2_ controlled incubator at 37°C and 5% CO_2_. As previously described (18) parasites where diluted 1:100 in pre-warmed complete Media (Gibco) and 5% Albumax (Sigma) without phenol red. 100 µL of this suspension was imaged either with, or without 100 µg/mL R1 inhibiting peptide in 35 mm glass bottom culture dishes (MatTek, Ashland, MA, USA). Image acquisition was performed with an AxioCam HRm camera (Zeiss) and Axiovision (Zeiss). Image software used was ImageJ.
